# Sexual and reproductive health considerations in the care of young adults with inflammatory bowel disease: A multidisciplinary conversation

**DOI:** 10.1016/j.hctj.2023.100033

**Published:** 2023-12-07

**Authors:** Sydney Reed, Amy K. Bugwadia, Sneha Dave, Hannah E. Wilson, Prathikka Ramesh, Hilary K. Michel

**Affiliations:** a Generation Patient, Crohn’s and Colitis Young Adults Network, USA; b Stanford University School of Medicine, USA; c Indiana University School of Medicine, Indianapolis, USA; d Saveetha Medical College Hospital, Chennai, Tamil Nadu, India; e Clinical Pediatrics - Nationwide Children’s Hospital and The Ohio State University College of Medicine, USA

**Keywords:** Sexual health, Reproductive health, Fertility, Young adults with inflammatory bowel disease, Adolescents with inflammatory bowel disease, Adolescents and young adults

## Abstract

The effects of inflammatory bowel disease (IBD) and the medications used to treat it on sexual and reproductive health can be significant, impacting the quality of life of patients across gender identities. This article presents insights from a roundtable discussion facilitated by the Crohn's and Colitis Young Adults Network (CCYAN) between young adult patients with IBD and medical professionals, including physicians, nurses, psychologists, and trainees/medical students. It underscores the distinction between sexual and reproductive health, emphasizing the need to address both aspects comprehensively. The discussion identified key themes, including sexual dysfunction and well-being in IBD patients; discussing sexual health with young adult IBD patients; current research in reproductive health: gaps and opportunities; discussing reproductive health with young adult IBD patients; and providing resources and comprehensive multidisciplinary care.

## Introduction

Sexual and reproductive health are distinct but equally important considerations in treating patients with inflammatory bowel disease (IBD). While discussions around sexual and reproductive health tend only to encompass the reproductive aspects, such as fertility and family planning, it is important to incorporate sexual health in these conversations, including socioemotional and physical well-being with regard to sexuality, sexual activity, and sexual relationships. Moreover, both sexual and reproductive health concerns often overlap with issues of body image, interpersonal relationships, and mental well-being, which can have a marked impact on an individual’s quality of life ^1^. Given the potential repercussions of unaddressed issues in these areas and the fact that more than 50% of IBD patients are diagnosed before the age of 35 ^2^, conversations focused on sexual and reproductive health between healthcare providers and young adult patients are of the utmost significance.

The Crohn's and Colitis Young Adults Network (CCYAN) is an international community and platform for young adults with IBD, working to address the unique needs of the adolescent and young adult (AYA) patient population. The CCYAN facilitated a roundtable discussion between young adult IBD patients and medical professionals, including physicians, nurses, psychologists, and trainees/medical students, to examine sexual health, reproductive health, and fertility in the care of young adults with IBD. This roundtable session began with a presentation by Dr. Alyse Bedell, Assistant Professor of Psychiatry and Behavioral Neuroscience and Assistant Professor of Medicine at the University of Chicago, and Rachael Whittemore, a young adult patient and physician assistant living with IBD. The discussion that followed centered on the following major themes: sexual dysfunction and well-being in IBD patients; discussing sexual health with young adult IBD patients; current research in reproductive health: gaps and opportunities; discussing reproductive health with young adult IBD patients; and providing resources and comprehensive, multidisciplinary care.

### Sexual health: sexual (Dys)function and sexual wellbeing in IBD patients

Sexual health is a vital component of overall well-being ^3^. IBD can have a profound effect on sexual health and function, notably impacting the quality of life for both women and men. Several studies have shed light on the prevalence and consequences of sexual dysfunction, defined as a disturbance in the sexual response cycle manifesting as decreased sexual desire, orgasm, and erectile dysfunction in individuals with IBD ^4^. Rates of sexual dysfunction are notably high among men and women with IBD, with women experiencing rates ranging from 50% to as high as 97%, particularly among newly diagnosed individuals 5, 6. One study found that the rates of sexual dysfunction in women with IBD were 61.9% as compared to 24.4% in healthy controls ^4^. Men with IBD also face a substantial burden, with the rate of erectile dysfunction being 43.5% in patients with IBD compared to 12.5% in healthy controls ^4^. Furthermore, specific subgroups within the IBD patient population, such as those with ostomies or perianal disease, face a further increased risk of sexual dysfunction 1, 4, 7.

During our roundtable discussion, one physician used the biopsychosocial model to explain the multifactorial causes of sexual dysfunction in IBD. This comprehensive approach considers biomedical, psychological, social, and interpersonal factors. Biomedical factors include disease activity, pain, surgical interventions, fatigue, pelvic floor dysfunction, and the side effects of medications used to manage IBD. Psychological factors such as stress, mood disorders, and trauma, all of which can be heightened in IBD, can also contribute to sexual dysfunction. Furthermore, sociocultural factors like religious beliefs, cultural norms, and access to sexual education, as well as interpersonal factors, including relationship dynamics and partner attitudes, play a considerable role in the experience of sexual dysfunction among IBD patients ^8^.

The effects of IBD on sexual well-being, function, and the perception of one's physical appearance are distinctly notable for young adults. Body image dissatisfaction can be exacerbated in people with IBD, especially those who are currently experiencing a disease flare or being treated with steroids, which can cause a variety of distressing symptoms, including (but not limited to) depression, weight gain, fluid retention, acne, and increased facial hair. 9, 10 These changes can feel devastating to adolescent and young adult IBD patients, who are at a stage of life characterized by physical development and the formation of critical interpersonal relationships, both of which contribute to increased attention to body image, amplifying the weight of IBD's impact on these facets of their lives.

### Discussing sexual health with young adults with IBD

When discussing the challenges surrounding conversations about sexual and reproductive health with young adult patients, both patients and providers brought up the difficulty presented when a parent or caregiver is in attendance. One patient shared a personal experience in which their parent interrupted a conversation regarding birth control and expressed their belief that the patient was not engaging in sexual activity or planning to get pregnant anytime soon, so it was unnecessary for the clinician to discuss contraception at that time. This patient emphasized the need for strategies to traverse such delicate situations where parents or guardians might preemptively shut down important conversations. One clinician shared their approach to addressing potential discomfort when discussing sensitive topics with parents or caregivers present by informing patients that the caregiver may be asked to leave the room at some point to create a private space for conversation. This strategy was affirmed by another provider who felt comfortable, as the physician, asking parents or caregivers to step out when needed, reinforcing her commitment to creating a safe and confidential space for patients to share sensitive information. Another patient suggested clinics incorporate questions related to sexual health and family planning on intake forms, thereby signaling the clinician's willingness to engage in these discussions while also normalizing such conversations within the healthcare setting.

Emphasis was also placed on the importance of healthcare professionals avoiding assumptions and using open-ended language when discussing sensitive topics, including sexual activity. One participating physician underscored the importance of broadening the scope of these conversations to be inclusive and beneficial to all patients. This includes not assuming whether or not a person is sexually active and whom the person is attracted to and/or having sex with. One best practice is to use neutral language when asking about the sex and gender of the patient’s sexual partner(s) (e.g., using the phrase “partner or partner(s)” instead of assuming the sex or gender of the partner or assuming the number of partner/partners). Additionally, if a patient does indicate that they are sexually active, follow-up questions about the sexual activity that the patient engages in can be phrased in an open, non-judgmental way (e.g., “Sex can look different for different people. I recognize that this can be an uncomfortable or awkward topic to discuss, but it is an important part of your overall health, and it can also help us ensure you get the appropriate follow-up health screenings. That being said, what kind of sexual contact/activity do you engage in? Examples include oral sex, vaginal sex, and anal sex.”). Asking these questions while contextualizing the relevance of these topics to a patient’s overall health (i.e., connecting it to safe and inclusive sexual health education, tailoring sexual health screenings depending on which body parts are involved) can be a way of showing solidarity and support for patients’ diverse experiences while also encouraging a more comprehensive understanding of sexual health.

Comprehensive sexual health education has an added layer of importance for individuals with IBD, as they face an elevated risk of contracting high-risk HPV infections and developing cervical neoplasia, potentially linked to immunosuppressant use 11, 12. Additionally, the resemblance of IBD symptoms to colorectal STDs poses a risk of delayed diagnosis ^13^. This underscores the importance of thorough patient education about sexual health, including practicing safe sex and undergoing regular sexual health screenings.

### Current research in reproductive health: gaps and opportunities

There are several notable gaps and needs for further research, largely regarding fertility, reproductive options, and the impact of IBD and its treatments on both. First and foremost, from the patient's perspective, there appears to be a lack of clarity and education about the role of IBD and IBD medications on contraceptive choice, family planning, and pregnancy ^14^. This ambiguity can leave patients with IBD uncertain about how their condition and its management might affect their fertility and reproductive choices. There was a consensus among roundtable participants that many individuals with IBD assume that they will automatically face fertility issues due to their condition, highlighting the need for accurate education and dispelling misconceptions surrounding fertility and IBD.

Additionally, given the continuous emergence of new data on the use of IBD medications in pregnancy, frequently revisiting conversations about reproductive health can provide an important opportunity for clinicians to share updated information with patients. Notably, multiple patient participants expressed concerns that there is insufficient data to support claims by medical practitioners regarding medication safety and impacts (or lack thereof) on sexual and reproductive health. This highlights the need for clinicians to stay up-to-date about the currently available data regarding the safety of certain medications and to provide this updated information to patients. Having these conversations is relevant not only when a patient switches therapies but also for patients who have been on certain therapies long-term since the reproductive health data and subsequent clinical recommendations may have changed since the initial treatment induction.

### Discussing reproductive health with young adults with IBD

Discussing reproductive health with young adult patients can help them make informed decisions for themselves now and in the future. Still, it is crucial to recognize that family planning is a deeply personal matter, and healthcare providers should always respect their patients' autonomy. No conversation about reproductive health should ever be forced; however, offering an opportunity to discuss these topics can have profound benefits. These conversations are often stigmatized, leaving patients with crucial information gaps, either because they are too shy to ask their clinician questions about reproductive health or because they are unaware that they can and should ask these questions.

One appreciable observation in the context of IBD is the higher rate of voluntary childlessness among IBD patients (13–19%) compared to the general population (6%) ^15^. Interestingly, IBD patients also tend to use contraceptives at higher rates, though the reasons behind this practice can be multifactorial. Some individuals with IBD use contraceptives not just for birth control but also to manage menstrual symptoms, which can be more severe in IBD patients ^16^. Roundtable patient participants brought up fears surrounding the role of inheritance of IBD, wondering whether choosing to have a biological child would predispose their child to the disease. Clinicians at the roundtable also shared a common assumption made by patients that, because they have IBD, they will be unable to have biological children. This further underscores the critical need for myth-busting and education to empower patients to make choices based on evidence-based information.

One young adult patient was told she “could not get pregnant” while on a specific medication, only to learn years later that this information was outdated, emphasizing the need for continuous, well-informed discussions between healthcare providers and patients. Specifically, when educating patients on the potential impacts of specific IBD medications, precise language is key: “difficulty getting pregnant” due to potential infertility is different from “should not get pregnant” due to potential medication impacts on the fetus. These distinctions are paramount to patients being thoroughly informed as a partner in their health, especially given the long-term impacts of this information on patient’s perception of their reproductive health and future conversations about family planning.

The lack of comprehensive sexual and reproductive health education calls attention to the importance of conversations between adult care providers and their young adult patients *after they transition from pediatric care*. This can be exceptionally relevant to patients diagnosed as children, especially those on certain long-term treatments. Given the age appropriateness and developmental considerations, their pediatric clinicians might not have had the opportunity to address the potential impacts of their treatment on reproductive health and fertility, or they may have had these conversations primarily with parents or caregivers and not specifically with the patient. In light of these circumstances, adult care clinicians should be cognizant of this gap and be prepared to incorporate these important discussions into their visits. This process begins by assessing the patient's existing knowledge, which can be achieved by asking open-ended questions to ascertain their understanding of their current treatments and any associated side effects, including those that might have implications for reproductive health. Subsequently, they can fill in information gaps as needed, ensuring that their patients are well-informed about the potential impacts of their treatment on their reproductive health and fertility moving forward.

For IBD patients with a uterus who are interested in discussing pregnancy and family planning, there are several crucial questions to consider. These include:

1. how long one should be in remission before attempting pregnancy, and what criteria (e.g., symptoms, labs, imaging, endoscopy/colonoscopy/pouchoscopy, etc.) the physician uses to determine whether the patient is in remission
2. safety of medications during pregnancy
3. special considerations for delivery (such as vaginal birth versus cesarean section)
4. post-delivery, including lactation, nutrient absorption, and potential health complications

One physician emphasized the importance of creating a structured approach to addressing pregnant patients or having questions about pregnancy to ensure the responses of all care team members are tactful and supportive. Rather than making assumptions, healthcare providers should ask questions to understand better the patient’s physical, mental, and emotional response to their pregnancy. An example of how to respond when a patient shares that they are pregnant, including the salient questions to ask and immediate recommendations for patients and clinicians, can be found in Appendix. The Appendix also includes a follow-up resource sheet that can provide newly pregnant patients with information about emotional health and pregnancy options.

### Providing resources and comprehensive, multidisciplinary care

An ever-present theme throughout our roundtable discussion was the need for easily accessible educational resources to empower patients in making informed decisions and effectively managing their sexual and reproductive health. Several physicians at our roundtable underscored this point and the importance of having reliable information, particularly considering the limited time available during medical appointments. Resources utilized by providers in the discussion are summarized in Table 1.

**Table 1:** Resources shared during the roundtable session.

Resource Title	Description
Exploring Reproductive Health Decision Experiences and Preferences of Women With Pediatric-Onset Inflammatory Bowel Diseases^14^	This study explored the reproductive health decision-making experiences and preferences of women diagnosed with IBD in the pediatric period to discover ways to improve this aspect of comprehensive care.
PIANO (Pregnancy Inflammatory bowel disease And Neonatal Outcomes) study^17^	The PIANO research study looks at the safety of IBD medications in pregnancy and short- and long-term outcomes of the children.
Inflammatory Bowel Disease in Pregnancy Clinical Care Pathway: A Report From the American Gastroenterological Association IBD Parenthood Project Working Group^18^	The goal of the IBD in Pregnancy Clinical Care Pathway is to provide guidance on the continuum of care and best practices for managing patients with IBD who are either pregnant or have a desire to become pregnant.
The American Association of Sexuality Educators, Counselors and Therapists (AASECT)^19^	The American Association of Sexuality Educators, Counselors and Therapists (AASECT) provides training, community and visibility to promote the understanding of human sexuality and healthy sexual behavior. The AASECT directory can be used to locate and identify AASECT Certified Educators, Counselors and Therapists for sexual education, counseling or therapy purposes.
Example of Pregnancy Phone Triage Note (Smartphrase: “. GIPregnancyTriage”) *This resource can be found in the Appendix of this paper*	This is a resource adapted from the Nationwide Children’s Hospital Center for Pediatric and Adolescent Inflammatory Bowel Disease and can be found in the Appendix of this manuscript. It includes questions to ask patients who disclose that they are newly-pregnant and immediate recommendations for patients and their clinicians. *Please note that this document is for educational purposes only and does not constitute medical advice. Recommendations may vary based on geographic location and local resources.*
Information Guide for Newly-Pregnant IBD Patients *This resource can be found in the Appendix of this paper*	This is a resource adapted from Nationwide Children’s Hospital Center for Pediatric and Adolescent Inflammatory Bowel Disease and can be found in the Appendix of this manuscript. It is a resource document to provide newly-pregnant patients and includes information about emotional health and pregnancy options. *Please note that this document is for educational purposes only and does not constitute medical advice. Recommendations may vary based on geographic location and local resources.*

Roundtable participants identified a multidisciplinary approach as essential to providing comprehensive care to individuals with IBD. This approach involves specialists from various fields, including gastroenterologists, gynecologists and obstetricians, sex therapists, surgeons, and physical therapists. In particular, sex therapy, a form of psychotherapy, was highlighted as a critical component in addressing sexual dysfunction in individuals with IBD. Participants recognized that GI psychologists are relatively rare, but certified sex therapists are more common and can work effectively with individuals who have medical conditions, including IBD.

Collaboration between healthcare providers, such as gastroenterologists and therapists, was encouraged to ensure that patients are connected with the right resources. This collaboration helps address not only the physical symptoms but the emotional and psychological impacts that can affect sexual health as well. Gastroenterologists can play a vital role in diagnostics and ensuring that patients are referred to appropriate specialists, particularly in cases involving perianal disease, fistulas, pelvic floor dysfunction, or other issues that may contribute to sexual dysfunction or discomfort.

## Conclusion

Addressing sexual well-being and reproductive health in the care of young adults with IBD is a complex but vital aspect of comprehensive care. The distinction between sexual and reproductive health has been emphasized, highlighting the need to address both aspects thoroughly. It is essential to recognize that the effects of IBD and the medications and surgeries used to treat it can have serious repercussions on a patient’s quality of life. As discussed, the prevalence and multifactorial causes of sexual dysfunction in IBD patients are a pressing concern. Addressing these issues requires effective, transparent communication between patients and members of their care team to ensure accurate education, dispel misconceptions surrounding fertility and medication safety, and create a safe environment in which patients feel comfortable asking questions and bringing up concerns relating to sexual and reproductive health. Strategies to create a safe and confidential space for these conversations have been discussed, emphasizing the importance of open-ended language and avoiding assumptions. Lastly, given the complexities in caring for individuals with IBD, having accessible educational resources and utilizing a multidisciplinary approach can help patients successfully navigate sexual health concerns and put them in the best possible position to make informed decisions regarding their reproductive health and family planning.

## CRediT authorship contribution statement

**Wilson Hannah E.:** Writing – review & editing. **Ramesh Prathikka:** Writing – review & editing. **Michel Hilary:** Writing – original draft, Writing – review & editing. **Dave Sneha:** Funding acquisition, Project administration, Writing – review & editing. **Bugwadia Amy:** Writing – original draft, Writing – review & editing. **Reed Sydney:** Formal analysis, Writing – original draft, Writing – review & editing.

## Data Availability

No data was used for the research described in the article.
