# Supplementary material for: Sexual and reproductive health considerations in the care of young adults with inflammatory bowel disease: A multidisciplinary conversation
Source: Health Care Transit. 2023 Dec 7;2:100033. doi: 10.1016/j.hctj.2023.100033 (PMC11658044; doi:10.1016/j.hctj.2023.100033)
Supplement: Supplementary file 1 — Supplementary material [file mmc1.docx]

**Appendix**

**Resource 1: Example of Pregnancy Phone Triage Note (“.GIPregnancyTriage”)**

*This resource has been adapted from Nationwide Children’s Hospital Center for Pediatric and Adolescent Inflammatory Bowel Disease. If you adapt and use this as a resource, please credit Nationwide Children’s with the following sentence:* “This resource has been adapted from Nationwide Children’s Hospital Center for Pediatric and Adolescent Inflammatory Bowel Disease.” *Additionally, please note that these documents are for educational purposes only and do not constitute medical advice. Recommendations may vary based on geographic location and local resources.*

Received incoming call that @FNAME@ is pregnant.

| Diagnosis | *** |
| --- | --- |
| Primary Gastroenterologist | *** |
| How did patient find out about pregnancy | {pregnancytest:31231} |
| Date of last menses | *** |
| Current IBD medications (esp. methotrexate, tofacitinib, upadacitinib) | *** |
| Do you have appt with OB/GYN? | {obgyndate:31232} |
| If <18 years old, is family aware? | {YES NO N/A:18019} |
| Best phone number for follow up | *** |
| Is it okay to leave message on preferred phone? | {YES/NO:11203} |
| Is it okay to send messages via MyChart? | {YES/NO:64} |
| Any additional questions/concerns | *** |

**Immediate recommendations for patient:**

1. Hold teratogenic medication (methotrexate)

2. Start prenatal vitamin

3. Follow up with other prescribers/clinics to evaluate safety of non-GI medications

**Recommendation for nurses:**

Notify team: Primary gastroenterologist

Involve other team members, if needed: psychology, social work, etc.

**Recommendations for providers:**

1. Order serum pregnancy test, if needed

2. Further evaluate current medications

3. Refer to adult GI provider

**Resource 2: Example of Information to Provide to Newly-Pregnant Patients**

*This resource has been adapted from Nationwide Children’s Hospital Center for Pediatric and Adolescent Inflammatory Bowel Disease. If you adapt and use this as a resource guide for your patients, please credit Nationwide Children’s with the following sentence:* “This resource has been adapted from Nationwide Children’s Hospital Center for Pediatric and Adolescent Inflammatory Bowel Disease.” *Additionally, please note that these documents are for educational purposes only and do not constitute medical advice. Recommendations may vary based on geographic location and local resources.*

**IBD and Pregnancy**

With good planning and care, patients with IBD can have healthy pregnancies and babies!

Informing your healthcare team of your pregnancy is an important first step so we can answer questions and help you navigate next steps:

- IBD Treatments

o With the exception of methotrexate, you should **continue taking your IBD medications** unless told otherwise by your healthcare team.

o If you are on medicine for something other than IBD, discuss with your healthcare team/pharmacist to make sure they are safe for you and baby.

- Medical Team Recommendations

o Since patients with IBD are at increased risk for some pregnancy complications, it is important to follow with a **high risk obstetrician (OB)** or **Maternal Fetal Medicine (MFM)** specialist for your prenatal care.

o We will also transfer your IBD care to an **adult IBD provider,** since they are experts in caring for pregnant patients with IBD. Your current pediatric IBD provider will place a referral and you will be contacted to schedule an appointment.

o It is very important to establish care as soon as possible and attend the first appointment with your new provider – they are unable to prescribe medications until you are seen.

- Need help finding an adult IBD provider, high risk OB, or other pregnancy-related resources? **Contact your IBD provider or nurse** to get you connected with the right folks.

**Helpful links about Pregnancy and IBD**

- [IBD Parenthood Project | Official Site (gastro.org)](https://ibdparenthoodproject.gastro.org/)
- [PIANO | Gastroenterology (ucsf.edu)](https://gastroenterology.ucsf.edu/research/piano)
- Crohn’s Colitis Foundation
  - [Family planning and IBD: Expert answers to common questions | Crohn's & Colitis Foundation (crohnscolitisfoundation.org)](https://www.crohnscolitisfoundation.org/family-planning-and-ibd-expert-answers-to-common-questions)
  - [PIANO Study offers hope and comfort to prospective and current IBD moms | Crohn's & Colitis Foundation (crohnscolitisfoundation.org)](https://www.crohnscolitisfoundation.org/blog/piano-study-offers-hope-and-comfort-to-prospective-and-current-ibd-moms)

**Emotional Health During Pregnancy**

It’s normal to have lots of thoughts and feelings when you learn you are pregnant. You may feel happy, excited, shocked, scared, worried, or a mix of these at the same time.

These websites can help you work through these thoughts and think about next steps:

- [I'm Pregnant, Now What? | Pregnancy Options For Teens (plannedparenthood.org)](https://www.plannedparenthood.org/learn/teens/stds-birth-control-pregnancy/i-think-im-pregnant-now-what)
- <https://www.plannedparenthood.org/learn/pregnancy/pregnancy-options>
- Support, which can come in many different forms, is especially important at this time. Try to identify at least 1 person in your life you can talk to as you go through this process.
- There are several organizations that can provide support as well:
- To find a mental health provider, contact your insurance provider (via phone or online) and search “in-network” mental health providers. Then, check the Psychology Today website (<https://www.psychologytoday.com/us/therapists>) to see if any of those providers have expertise in what you are looking for (ex. postpartum, telehealth options, etc.).
- All-Options
  - Free hotline that provides a confidential space to talk about making decisions about a pregnancy
  - Specific resource pages, including topics of pregnancy, parenting, abortion, and adoption
  - 1-888-493-0092 (M-F 10am-1am; Sa-Su 10am-6pm)
  - <https://www.all-options.org/find-support/talkline/>
- Pregnancy Options Workbook
  - https://www.pregnancyoptions.info/pregnancy-options-workbook
- Exhale Pro-Voice
  - A text line that offers peer counseling for people who have had abortions and their loved ones, and training on how to provide support after abortion
  - <https://exhaleprovoice.org/>

***If you’re experiencing a crisis or would like to talk to someone now about your mental health, please utilize any of the following supports:***

- National Maternal Mental Health Hotline: 1-833-943-5746 (for support before, during, and after pregnancy from professional counselors; English and Spanish)
- National Suicide and Crisis Lifeline: Call 988
- National Suicide Prevention Lifeline: 1-800-273-8255 (suicidepreventionlifeline.org)
- National Crisis Text Line: Text "4HOPE" at 741-741
- Go to the nearest Emergency Department

**New Pregnancy: Know Your Options**

**Have and Raise the Baby:**

- If you choose to keep your pregnancy, you will require routine prenatal care with an Obstetrician (OB)/Gynecologist (GYN) or Midwife. Prenatal care is an important part of staying healthy during pregnancy.
- For more information on prenatal care, please visit the American College of Obstetricians and Gynecologists (ACOG) website: acog.org

**Adoption:**

- There are 2 types of adoption:
  - *Open adoption:* You and the adoptive parents may exchange information about each other, so the baby will know your identity. However, there is no single definition of what an open adoption is. Open adoptions can vary widely in the types and amount of contact shared between parties.
  - *Closed adoption:* Neither you nor the adoptive parents know the identity of the other, but important medical and sexual history may be shared.
- You can arrange your plan for an adoption through a local adoption agency. Please ask your OB/GYN if you need support in finding a local adoption agency.
- For more information on adoptions, please visit: americanadoptions.com

**Terminate the Pregnancy:**

- You can end a pregnancy via a medical or surgical route, though your options vary depending on how far along you are in your pregnancy, state laws. Minors need parental consent or court authorization to get an abortion.
- For more information on abortions, please visit: plannedparenthood.org
- For more information on up-to-date abortion laws in your state, or to find a local facility that can provide abortion services, please visit: abortionfinder.org
- American Psychological Association article on abortion and mental health (see above for mental health resources):<https://www.apa.org/monitor/2022/09/news-facts-abortion-mental-health>
